# Ultrasound-Guided Proximal Radial, Ulnar, Median and Musculocutaneous (RUMM) Nerve Block Technique in Rabbit (*Oryctolagus cuniculus*) Cadavers: Medial vs. Lateral Approach †

**DOI:** 10.3390/ani15030294

**Published:** 2025-01-21

**Authors:** Giulia Teotino, Ricardo Felisberto, Derek Flaherty, Hamaseh Tayari

**Affiliations:** Southern Counties Veterinary Specialists, Forest Corner Farm, Ringwood, Hampshire BH24 3JW, UK; giulia.teotino@gmail.com (G.T.); derek.flaherty@scvetspecialists.co.uk (D.F.); hamasehtayari@gmail.com (H.T.)

**Keywords:** rabbits, locoregional anaesthesia, proximal RUMM block, ultrasound

## Abstract

This prospective, experimental, randomised, assessor-blinded cadaveric study was undertaken to describe the sono-anatomical features of the radial, ulnar, median and musculocutaneous (RUMM) nerves in rabbits and to develop and evaluate an ultrasound (US)-guided proximal RUMM block technique comparing a medial versus a lateral approach. A total of 13 adult rabbit cadavers were used. In Phase I of the study, four cadavers were used for anatomical dissection and to design and test a lateral and medial single injection point US-guided proximal RUMM block technique, while in Phase II, the medial and lateral approaches were randomly performed on nine cadavers administering 0.1 mL kg^−1^ injectate. After dissection, nerve staining was categorised as adequate (all nerves stained ≥4 mm) or inadequate (at least one nerve not stained or stained <4 mm). Staining spread was compared with Fisher’s exact test, with *p* < 0.05 considered statistically significant. From Phase I, the axillary fascia containing all RUMM nerves was identified. The radial nerve exited the fascia right after the humeral head. In the lateral approach, the transducer was angled at 80° to the humerus longitudinal axis. In the medial approach, the transducer was placed perpendicularly to the humerus longitudinal axis. In both approaches, the brachial artery appeared as a rounded and anechoic structure, the musculocutaneous nerve as hypoechoic and oval and the radial nerve as a honeycomb, and the ulnar and median nerves were identified adjacent to each other. The radial nerve was selected as the injection point for both approaches using an in-plane technique. In Phase II, the injectate was found outside the axillary fascia in zero out of nine and five out of nine thoracic limbs, with an adequate staining in nine of nine and two of nine injections (*p* < 0.01) using the medial and lateral approach, respectively. Thus, a US-guided proximal RUMM block technique is feasible in rabbits, and the medial approach demonstrated evidence of a more consistent stain of the RUMM nerves.

## Simple Summary

After dogs and cats, rabbits are the third most common pet kept in the UK, and they are often presented to veterinarians for a variety of surgical procedures. Many anaesthetic and pain-relieving drugs—in particular, opioids—can have adverse effects in this species, leading to prolonged recovery from general anaesthesia and gastrointestinal status, which may contribute to subsequent patient death; this is affirmed by the markedly increased anaesthetic mortality in rabbits compared to dogs and cats. Consequently, achieving a reduction in anaesthetic or opioid requirements is likely to lessen morbidity and mortality in this species. Locoregional analgesia techniques (“nerve blocks”) involve the injection of local anaesthetic drugs to prevent the transmission of pain sensation to the brain, effectively “numbing” the affected area. By doing this as part of an anaesthetic procedure, it allows a marked reduction in both anaesthetic and opioid requirements. This study was undertaken to assess the feasibility of one specific locoregional technique (RUMM block) of the front leg in rabbit cadavers and demonstrated that this could be successfully performed; therefore, it may be of value in reducing complications in rabbits undergoing procedures on their forelimbs.

## 1. Introduction

Peri-anaesthetic mortality is greater in rabbits (1.39%) compared to dogs (0.17%) and cats (0.24%) [1]. Deaths are mainly attributed to prolonged recovery from anaesthesia, untreated pain and post-operative ileus [1,2,3,4]. Post-operative pain reduces food intake and motor and hopping activity [5,6], which may contribute to the higher peri-anaesthetic mortality reported in this species [1,2].

The implementation of regional anaesthetic (RA) techniques has been associated with a lower mortality rate in dogs [7]. Although the same has not yet been confirmed in rabbits, there has been an increased interest in developing new RA techniques in this species [8,9,10,11]. In rabbits, RA techniques reduce systemic analgesic drug requirements and possible side effects such as hypothermia, hypoventilation and post-operative ileus [3,12,13,14].

In dogs, the radial, ulnar, median and musculocutaneous (RUMM) nerves can be effectively blocked through a medial approach and a single proximal in-plane injection ultrasound (US)-guided technique [15,16]. Conversely, in cats, the proximal RUMM block has been tested using either a medial or lateral approach [17]. However, the effectiveness of this technique depends on the ability to inject the local anaesthetic within the axillary fascia, where all four nerves are contained simultaneously.

To the best of our knowledge, it is currently unclear whether the axillary fascia is present and can be detected echographically in rabbits. Hence, the aim of this study, conducted using rabbit cadavers, was to achieve the following:

(1) Investigate the anatomy and the sono-anatomical features of the axillary region, with a specific emphasis on the layout of the RUMM nerves.
(2) Develop two US-guided proximal RUMM block techniques utilising a single injection point and an in-plane needling technique, one for medial and one for lateral approaches.
(3) Compare the quality of the US visualisation and nerve staining achieved with both approaches.

To the authors’ knowledge, there are no studies supporting the superiority of the medial vs. lateral proximal RUMM block approach; therefore, it was expected that both would be viable. Based on this, the initial hypothesis was that there would be no difference in the number of nerves stained or the length of staining between the two approaches.

## 2. Materials and Methods

### 2.1. Animals

This study was performed at Southern Counties Veterinary Specialists (Ringwood, UK) in February 2024, following the Anatomical Quality Assurance (AQUA) checklist [18], and included a total of 13 cadavers of adult rabbits (*Oryctolagus cuniculus*), 4 cadavers for Phase I and 9 cadavers for Phase II.

No animals were euthanised for the purpose of this study; therefore, no ethical committee approval was obtained. The rabbits were obtained frozen from a certified seller of reptile food (Kiezebrink UK Limited, Great Chesterford, UK). The carcasses were gradually thawed for 48 to 72 h in a dedicated room at a controlled temperature (22 °C) (Southern Counties Veterinary Specialists, Ringwood, UK) before the beginning of each phase of the study. Any cadaver with anatomical alterations in the area of interest for the study was excluded. Each cadaver was assigned a unique identification number, and sex, weight and intramuscular temperature were recorded. The temperature was recorded using a thermometer probe (Meat thermometer, Umedo 2, Cardiff, UK) placed in the vastus lateralis muscle, the largest part of the quadratus femoris muscle. Additionally, a body condition score was assigned using a UK Pet Food’s Rabbit Size O-Meter (https://www.ukpetfood.org/resource/rabbit-size-o-meter.html accessed on 4 February 2024).

### 2.2. Phase I: Sono-Anatomical Study of the Brachium, Development and Pilot Investigation of the Proximal RUMM Block Techniques (Lateral and Medial Approaches)

The first cadaver was used for gross anatomical dissection of the axillary area to familiarise the investigators with the anatomical structures. With the cadaver positioned in dorsal recumbency, both thoracic limbs were dissected along the medial aspect. The skin, pectoralis muscles and the latissimus dorsi muscle were carefully removed to reveal the axillary area containing the radial, ulnar, median and musculocutaneous nerves. The length of each of the four nerves was measured from their emergence at the level of the brachial plexus to their more distal visible point at the level of the elbow.

Another cadaver was used to perform the sono-anatomical study of the axillary area and two others (four thoracic limbs) to design the lateral and the medial single injection, in-plane needling, US-guided proximal RUMM block technique. A total volume of 0.1 mL kg^−1^ of lidocaine 2% (Lidocaine, Hameln, Pharmaceuticals, Glouchester, UK) mixed with a tissue dye solution (Tissue marking dye yellow, Mopec, Madison Heights, MI, USA) in a 3:1 *v:v* ratio was injected using a single injection site. The volume was chosen based on a previous publication where 0.05 mL kg^−1^ and 0.1 mL kg^−1^ of the injectate were compared to perform a saphenous nerve block in rabbits [8]. A veterinary dedicated ultrasound system (Fujifilm Sonosite Inc. SII Veterinary Ultrasound System, Bothell, WA, USA) with a linear probe (L25x,13-6 MHz Linear transducer Bothell, WA, USA) was used. The skin of the axillary, brachial and thoracic regions was clipped and scrubbed. An ultrasound gel (Aquasonic 100, Parker, Fairield, NJ, USA) was applied on the rabbit’s skin to allow optimal contact with the probe.

**Figure 1:**
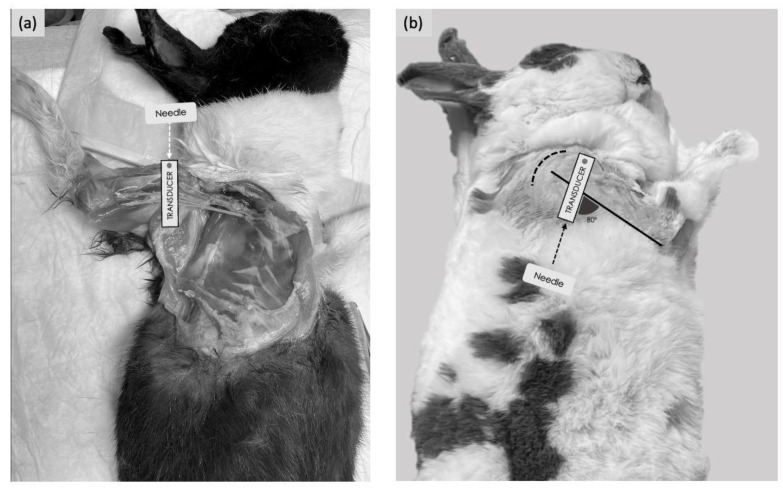
Positioning of the animal, transducer orientation and needling technique used to perform the ultrasound-guided RUMM block with a medial or lateral approach. (**a**) Medial approach: the transducer was placed over the head of the humerus perpendicularly to the humeral axis. An in-plane needling technique was performed with a cranio-caudal direction. (**b**) Lateral approach: the transducer was placed over the head of the humerus; the internal angle created between the probe and the longitudinal axis of the humerus, with the lateral approach, was approximately 80°. An in-plane needling was performed with a caudo-cranial direction. The dashed curved line represents the shoulder joint.

For the medial approach, the rabbits were placed in dorsal recumbency with the thoracic limb gently abducted laterally, and a cushion was positioned underneath the shoulder to allow good contact between the US probe and the skin. Starting from the medial aspect of the elbow, the US probe was moved proximally up to the axillary fossa to localise the optimal acoustic window (defined as all four nerves visible within the axillary fascia). This was obtained with the transducer positioned immediately distal to the shoulder joint, perpendicular to the long axis of the humerus, with the marker facing cranially (Figure 1a). For the lateral approach, the rabbits were positioned in lateral recumbency, with the thoracic limb to be injected uppermost. A cushion was placed between the thoracic limbs to allow positioning of the humerus of interest parallel to the table. Starting from the elbow, the US probe was moved proximally up to the humeral head. The internal angle created between the probe and the longitudinal axis of the humerus was approximately 80° (Figure 1b).

The optimal acoustic window was identified with the transducer positioned on the lateral aspect of the tricipital region with the marker at the level of the humeral head and facing cranially.

On the optimal acoustic window for each approach, the distance from the skin to the most superficial part of the axillary fascia was measured.

Thereafter, the most convenient injection point for the medial and lateral approaches using the in-plane technique was simulated and determined in all thoracic limbs during this phase.

### 2.3. Phase II: Comparison Between Lateral and Medial US-Guided Proximal RUMM Techniques

A block technique randomisation (1:1) using lottery methodology was used to decide the limb to be injected (left or right) and the approach to be used (medial or lateral) for each of the remaining nine rabbit cadavers. A blind raffling was made to assign one of the two operators (both with expertise in proximal RUMM block execution; >50 proximal RUMM blocks performed in other species) to each cadaver. Each operator (*n* = 2) performed the same number of proximal RUMM block techniques and a similar number of approaches (operator 1: 5 lateral and 4 medial vs. operator 2: 4 lateral and 5 medial).

The acoustic window obtained for each thoracic limb was graded as excellent if all the RUMM nerves were visualised, good if the radial, ulnar and median nerves were visualised and poor if only the radial nerve was visualised [17].

All proximal RUMM block approaches were performed with a 21-gauge, 50 mm echogenic insulated needle (Echoplex, Vygon, Swindon, UK) connected to a 1 mL syringe (BD Plastipak, Becton Dickinson, Wokingham, UK) and primed with the lidocaine–dye solution as designed in Phase I of the study. For each US-guided proximal RUMM block technique performed, the distance from the skin to the most superficial part of the axillary was measured after the identification of the optimal US acoustic window. Immediately after the injection was performed bilaterally, anatomical dissection was carried out by another operator unaware of the approach used.

All nine rabbit cadavers were placed in dorsal recumbency, and after removing the skin, dissection was performed. The pectoralis and the latissimus dorsi muscles were removed from their insertion, and then the humerus was abducted to expose the neurovascular bundle. Subsequently, for each nerve, the presence or absence of staining was recorded, and, where present, the length of staining was measured with a standard ruler. The staining obtained was then classified as adequate if all four nerves were covered with tissue dye for a length ≥4 mm and as inadequate if <4 mm [19]. The percentage of staining was then calculated with respect to the total nerve length detected in Phase I.

### 2.4. Statistical Analysis

Statistical analysis was performed using R-programming language (https://cran.r-project.org/bin/macosx/ accessed 2 April 2024). The Shapiro–Wilk test was used to test the normality of the data. The results are presented as mean ± standard deviation or median (range), accordingly. The categorical data obtained during the dissection of Phase II were compared using the Fisher’s exact test. For that purpose, two groups, based on the approach tested (medial or lateral), were analysed based on two categories, adequate or inadequate, to determine if a statistically significant difference between the approaches was present. Significance was considered if *p* < 0.05.

## 3. Results

All the cadavers were in satisfactory conditioning after thawing and no significant damage was observed; therefore, all 13 rabbits were included in the study. For the nine carcasses (18 thoracic limbs) used to conduct Phase II, the mean intramuscular temperature was 22.9 ± 0.4 °C and the mean carcass weight was 2.5 ± 0.3 kg; six of the nine rabbits were male. The median (range) body condition score was 3 (3–4) out of 5.

### 3.1. Phase 1

**Figure 2:**
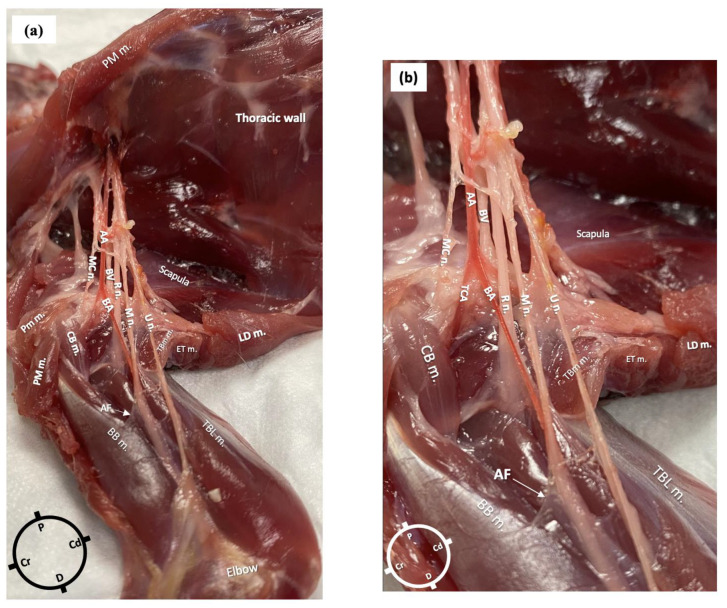
Gross anatomical dissection of the right axillary area of a rabbit in dorsal recumbency. The skin, the pectoralis muscles and the latissimus dorsi muscle were carefully removed to reveal the axillary area, which following careful dissection allowed the identification of the radial, ulnar, median and musculocutaneous nerves (axillary fascia was removed, but a small portion is still visible). (**a**) AA: axillary artery; BA: brachial artery; BV: brachial vein; R n: radial nerve; U n: ulnar nerve; MC n: musculocutaneous nerve; M n: median nerve; PM m: pectoralis major muscle; CB m: clavobrachialis muscle; LD m: latissimus dorsi muscle; AF: axillary fascia; BB m: biceps brachii muscles; TBL m: triceps brachii long head muscle; TM m: teres major muscle. (**b**) Magnified picture of Figure 1a to show the neurovascular bundle in more detail.

In the first cadaver, the anatomical dissections revealed the presence of an axillary fascia, a continuation of the paravertebral fascia, as a collection of connective tissue surrounding the nerves and vessels. The fascia was opened to expose the neurovascular bundle. The axillary artery branched at the level of the proximal head of the humerus into the transverse cubital artery and brachial artery. In relation to the brachial artery, the musculocutaneous nerve was located cranially, the radial nerve laterally and medially and the ulnar and the median nerves caudally. The radial nerve lay at the medial aspect of the humeral head from where it pierced the fascia to move towards the lateral aspect of the limb. At the level of the head of the humerus, a musculocutaneous nerve branch left the original nerve and the axillary fascia to supply the biceps brachii muscle (Figure 2a,b).

The lengths of the nerves, measured from their emergence at the level of the brachial plexus to their more distal visible point at the level of the elbow in the first rabbit, were as follows: for the radial nerve, 5 cm on the left limb and 4.5 cm on the right, for both the median and ulnar nerves, 9 cm bilaterally, and for the main musculocutaneous nerve, 4 cm on both limbs.

In the second cadaver, the lateral sonogram revealed that the proximal humerus was identified as a hyperechoic semicircle with its acoustic shadow located medially and cranially to the biceps brachii muscle. The axillary fascia was identified as a hyperechoic irregular structure containing the neurovascular bundle. The measured distance of the lateral aspect of the axillary fascia from the skin was 1 ± 0.2 cm.

For the medial sonogram, the proximal humerus was identified as an irregular acoustic shape, and its acoustic shadow was located laterally and cranially to the pectoralis muscles. The axillary fascia was identified as a hyperechoic structure with a regular lobulate shape containing the neurovascular bundle. The measured distance of the medial aspect of the axillary fascia from the skin was 0.59 ± 0 cm.

In both approaches, the brachial artery appeared as a rounded anechoic structure situated in the middle of the neurovascular bundle within the axillary fascia. The musculocutaneous nerve was identified as a hypoechoic flattened oval structure with a bright hyperechoic rim located cranially to the brachial artery. This nerve appeared to move towards the skin and away from the neurovascular bundle to innervate the proximal biceps muscle. The radial nerve was identified as a honeycomb structure, positioned caudo-laterally to the brachial artery on the medial approach and cranio-laterally in the lateral approach. The ulnar and median nerves were always identified in close proximity to each other and caudally to the brachial artery.

**Figure 3:**
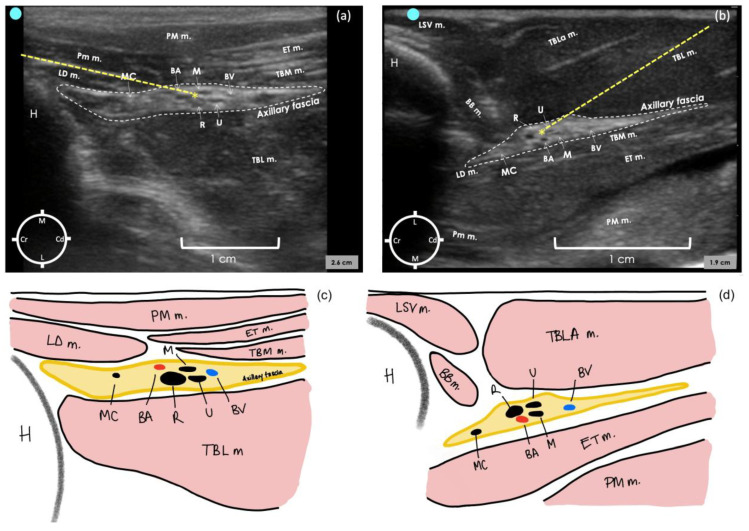
Ultrasound image of axillary region in a rabbit cadaver to perform the proximal radial, ulnar, median and musculocutaneous (RUMM) nerve block technique with a medial approach (**a**) and a lateral approach (**b**). Schematic illustration of the structures observed in (**a**,**c**) and (**b**,**d**). For the medial approach, the transducer was positioned immediately distal to the shoulder joint, perpendicular to the long axis of the humerus, with the marker facing cranially. For the lateral approach, the transducer was positioned on the lateral aspect of the tricipital region with the marker at the level of the humeral head and facing cranially. The internal angle created between the probe and the longitudinal axis of the humerus, with the lateral approach, was approximately 80°. BA, brachial artery; BV, brachial vein; R n, radial nerve; U n, ulnar nerve; MC n, musculocutaneous nerve; M n, median nerve; BB, biceps brachii muscle; PM m, pectoralis major muscle; LD, latissimus dorsi; TBL m, triceps brachii long head muscle; TBLa, triceps brachii lateral head muscle; TM m, teres major muscle; LVS m, levator scapulae ventralis muscle; ET m, epitrochlearis muscle; * injection point; dashed line, needle path.

In the last two cadavers, the radial nerve was selected as a target point to perform the proximal RUMM block technique with a single injection. This decision was based on its substantial size, which facilitates visualisation, and primarily because it is the first nerve to exit the fascia. Failing to target the radial nerve would lead to inadequate block in a live rabbit. The most convenient injection point was determined as targeting the proximo-medial aspect of the radial nerve for the medial approach and the proximo-lateral aspect of the radial nerve for the lateral approach (Figure 3a,b). This could be achieved by inserting the needle in plane to the transducer and advancing it cranio-caudally for the medial approach and caudo-cranially for the lateral approach. After the injection of the dye–lidocaine mixture in the last two cadavers, the medial approach resulted in all four nerves stained for a length of 2–4 cm in both limbs. In contrast, the lateral approach stained all four nerves for a length of 0.3–1 cm in one limb, while in the other limb, three out of four nerves were stained for a length of 0.4–1.2 cm.

### 3.2. Phase 2

Each of the two operators performed the same number of proximal RUMM block techniques and a similar number of approaches (operator 1: five lateral and four medial vs. operator 2: four lateral and five medial).

The quality of the US acoustic window was considered excellent in nine out of nine thoracic limbs assigned to the medial approach. Using the lateral approach, the quality of the US acoustic window was considered good in six out of nine thoracic limbs and poor in the remaining three (*p* < 0.01). The axillary fascia was visualised for the medial and the lateral approach at 0.6 ± 0.1 and 1.1 ± 0.2 cm from the skin, respectively.

**Figure 4:**
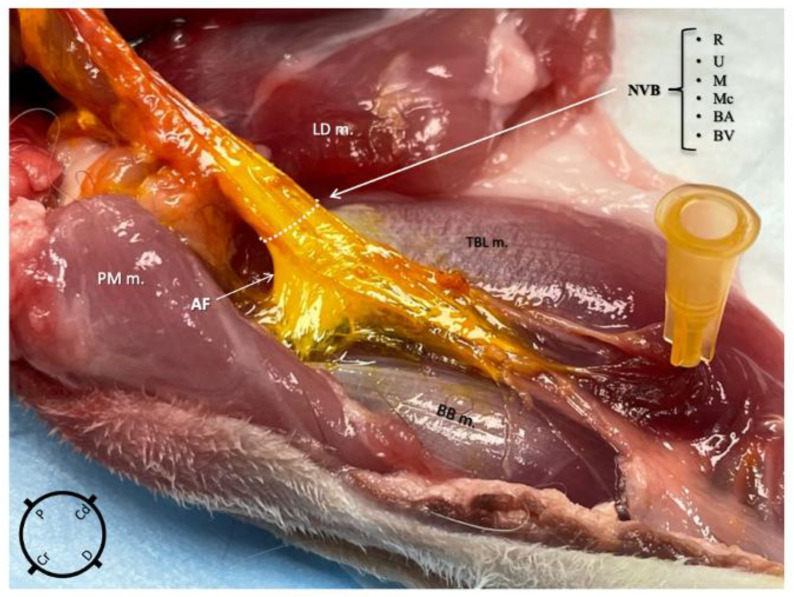
Anatomical dissection of the right thoracic limb of a rabbit, medial view, after an ultrasound-guided proximal radial, ulnar, median and musculocutaneous (RUMM) nerve block technique was performed using 0.1 mL kg^−1^ of yellow tissue dye solution with lidocaine 2% mixed in a 1:3 *v*:*v* ratio. The injectate is localised only within the axillary fascia staining the neurovascular structures (R, radial nerve; U, ulnar nerve; M, median nerve; Mc, musculocutaneous nerve; BA, brachial artery; BV, brachial vein). NVB, neurovascular bundle; BB m, biceps brachii muscle; PM m, pectoralis major muscle; LD m, latissimus dorsi muscle; TBL m, triceps brachii long head muscle.

Using the medial approach, the dye solution was successfully administered within the axillary fascia in nine out of nine thoracic limbs and no staining was found in the surrounding structures in any of them (Figure 4). Using the lateral approach group, the dye solution was successfully administered within the axillary fascia in eight out of nine thoracic limbs and the surrounding structures were stained in five out of nine. The difference in the occurrence of injectate found outside the fascia between the two approaches was statistically significant (*p* = 0.03). Following the staining, a thorough macroscopic inspection of the nerves revealed no evidence of gross structural damage or disruption.

The staining of the four nerves was always ≥4 mm (i.e., adequate) using the medial approach, while it was considered adequate in only two out of nine thoracic limbs using the lateral approach (*p* < 0.01). Of the remaining seven thoracic limbs, three nerves were stained in two of those, two nerves were stained in one of those and one nerve was stained in three of those.

For each approach, the number of nerves visualised in the acoustic window, the length of nerves stained and the percentage of staining obtained (based on the total nerve length reported in the results of Phase I) are reported in Table 1.

## 4. Discussion

The present study successfully developed a US-guided proximal RUMM block technique using a single injection point in rabbits. Contrary to our initial hypothesis, the medial approach was found to be significantly more consistent in staining than the lateral one, with the latter demonstrating greater difficulty in nerve visualisation, particularly the musculocutaneous nerve, and hence inferior accuracy in nerve staining. In contrast, the medial approach with an in-plane needle insertion and a total injectate volume of 0.1 mL kg^−1^ allowed consistent staining of the four nerves responsible for the innervation of the antebrachium. Therefore, it has the potential to produce desensitisation of most of the thoracic limb in rabbits, which could be useful as part of a multimodal analgesia approach in this species.

The present study is also the first to describe, similarly to dogs and cats, the presence of the axillary fascia in rabbits where all the RUMM nerves are contained [15,17,20].

In cats, a lateral US-guided axillary RUMM block performed with caudo-cranial needling but with multiple injection points (one at the level of the musculocutaneous nerve and one at the level of the radial nerve) successfully stained the four nerves [17]. Splitting the injectate in a similar manner, it is possible that the lateral US-guided proximal RUMM block technique designed here for rabbits would also yield a consistent nerve staining of all nerves; however, to decrease the risk of potential complications associated with multiple injections, this option was not considered.

A different needling orientation could have also been used for the lateral US-guided proximal RUMM block technique, as previously reported in cat cadavers [20]. According to these authors, the cranio-caudal needling orientation enhanced the staining rate of the musculocutaneous nerve. This needle orientation was considered during Phase I of this current study, but because the presence of the greater tubercle of the humerus made it challenging to guide the needle within the axillary fascia without bending it, it was not considered for Phase II.

A greater percentage of rabbit cadavers assigned to the lateral approach exhibited a poor sonogram image quality; interestingly, while the radial nerve was always sonographically visible, the musculocutaneous nerve was visible inside the axillary fascia only when the probe was positioned further distally.

While in dogs the radial nerve leaves the axillary fascia at the level of the first third of the humerus [15] and in cats at the mid humerus [17], based on our anatomical dissections, the radial nerve in rabbits exits the fascia immediately after the humeral head, which prevented us designing a lateral approach with the probe placed more distally.

Furthermore, a rabbit’s body weight resembles that of cats, but rabbits usually have shorter humeri [21]. This required a moderate abduction of the limb for the medial approach to allow adequate visualisation of the neurovascular bundle inside the axillary fascia. Despite potentially altering the usual anatomical relationship between nerves and vessels [22], the abduction of the limb facilitated adequate identification of all the target structures. This finding agrees with previous studies that utilised the medial approach for the proximal RUMM block, which also applied mild abduction [15].

A clearer view of all the nerves obtained with the medial approach and the use of a single injection point technique might increase the overall safety of the technique in live rabbits, reducing the risk of complications associated with multiple injections [23].

Furthermore, the number of nerves stained, as well as the length of staining of each individual nerve, was superior when the medial approach was used.

The volume of injectate used in our study, 0.1 mL kg^−1^, was selected to prevent excessive spread of the injectate outside the axillary fascia and to minimise the potential toxicity of local anaesthetics if applied to live animals, the threshold of which remains unknown for rabbits. It is worth considering that using a larger injectate volume, such as 0.15–0.2 mL kg^−1^ [15,17,20], might have resulted in better nerve staining and consequent performance of the technique with the lateral approach; however, nerve visualisation was still inferior with the lateral approach. Previous studies in cats [17] used a total volume of 0.15 mL kg^−1^ to perform the proximal RUMM block with the lateral approach and revealed optimal nerve staining with the dye solution.

It is not possible to exclude that the axillary fascia distended due to the mild abduction of the limb when using the medial approach, thereby facilitating the distribution of the injectate and, therefore, the spread obtained, but, if so, this could be considered a “desirable” consequence in the live animal.

The decision to mix lidocaine with the tissue dye was made to simulate the spread obtained when local anaesthetics are injected in a clinical scenario [24]; however, it is possible that the spread we observed in our cadavers is not entirely equivalent to that seen with “pure” local anaesthetic distribution administered in vivo.

The main challenge encountered in developing and performing the proximal RUMM block technique in rabbit cadavers was to achieve adequate visualisation of all the four nerves within the axillary fascia while ensuring sufficient space for needle insertion. Using the lateral approach, the quality of the visualisation was poorer than the medial approach probably because of the deeper position of the neurovascular bundle. In live rabbits, visualisation might be less problematic, as the presence of additional landmarks, such as the pulsatile brachial artery and distended brachial vein, would help to identify the target point (radial nerve). These landmarks, once visualised within the US window, should facilitate the localisation of the neurovascular bundle surrounded by the axillary fascia, thus allowing identification of the appropriate injection point.

This study has some limitations. Firstly, the echogenicity of the tissues could have been altered by freezing and thawing, potentially leading to discrepancies in accurately representing the sono-anatomy of live rabbits.

Secondly, the spread of the injectate inside the axillary fascia and the resistance encountered to injection can be different from that in live animals [25]. Although the mean intramuscular temperature of the cadavers was 22.9 °C, this is considerably lower than a live rabbit’s mean body temperature, and because the viscosity of a solution increases as the temperature decreases, this may have influenced the final spread of the injectate obtained.

The cadavers were only warmed up to room temperature and not to their in vivo body temperature to avoid excessive cadaveric decomposition, which could further interfere with the ultrasound image quality. Although, this may be a limitation to this study, most cadaveric studies use cadavers thawed to room temperature [26].

Thirdly, two experienced operators performed all the proximal RUMM block techniques. While this might have influenced the variability in the results obtained, their experience may have possibly masked difficulties in recognising different structures. Further studies involving operators with different training levels are warranted.

Finally, the injection pressure was not measured or standardised, and excessive pressure could have been generated on injection, potentially leading to axillary fascial rupture (particularly fragile in cadavers), resulting in the injectate leaking outside the axillary fascia, an event observed more frequently when the lateral compared to the medial approach was utilised.

## 5. Conclusions

The RUMM nerves in rabbits are all contained within the axillary fascia, which is clearly visible sonographically. The medial approach to the US-guided proximal RUMM block technique was superior in adequately staining all the RUMM nerves, without spreading of the injectate to the surrounding structures outside the axillary fascia. Despite these promising results, further work is needed in live rabbits to fully assess the analgesic efficacy of this technique.

## Figures and Tables

**Table 1:** Evaluation of the extent of nerve staining obtained by performing an ultrasound-guided proximal radial, ulnar, median and musculocutaneous (RUMM) nerve block by either medial or lateral approaches using 0.1 mL kg^−1^ volume of a dye solution (permanent yellow tissue dye in lidocaine 2% [1:3]) in nine rabbit cadavers (18 thoracic limbs). The frequency of nerve visualisation and adequate nerve staining obtained (length ≥ 0.4 cm) in each of the 9 limbs are reported. The percentage of nerve staining and the mean ± SD and range values of the nerve length staining are reported.

	Frequency of Nerve Visualisation	Adequate Staining Obtained	% of Nerve Stain	Mean ± SD (cm)	Range (cm)
Medial Approach					
Ulnar	9/9	9/9	34	2.6 ± 0.9	1.5–4
Median	9/9	9/9	34	2.6 ± 0.9	1.5–3.9
Musculocutaneous	9/9	9/9	38	1.5 ± 0.6	0.4–2.2
Radial	9/9	9/9	46	2.1± 0.7	1.4–3.5
Lateral Approach					
Ulnar	6/9	4/9	13	0.7 ± 0.9	0–2.2
Median	6/9	4/9	11	0.6 ± 0.9	0–1.8
Musculocutaneous	0/9	2/9	8	0.3 ± 0.7	0–1.7
Radial	9/9	6/9	19	0.9 ± 0.8	0–2

## Data Availability

The data present in this study are available within the article.
